# The Androgen Hormone-Induced Increase in Androgen Receptor Protein Expression Is Caused by the Autoinduction of the Androgen Receptor Translational Activity

**DOI:** 10.3390/cimb44020041

**Published:** 2022-01-25

**Authors:** Tiziana Siciliano, Ulrich Sommer, Alicia-Marie K. Beier, Matthias B. Stope, Angelika Borkowetz, Christian Thomas, Holger H. H. Erb

**Affiliations:** 1 Department of Urology, Technische Universität Dresden, 01307 Dresden, Germany; Tiziana.siciliano@tu-dresden.de (T.S.); AliciaMarie.Beier@uniklinikum-dresden.de (A.-M.K.B.); angelika.borkowetz@uniklinikum-dresden.de (A.B.); christian.thomas@uniklinikum-dresden.de (C.T.); 2 Institute of Pathology, Universitätsklinikum Carl Gustav Carus Dresden, 01307 Dresden, Germany; ulrich.sommer2@uniklinikum-dresden.de; 3 Mildred Scheel Early Career Center, Department of Urology, Medical Faculty and University Hospital Carl Gustav Carus, Technische Universität Dresden, 01307 Dresden, Germany; 4 Department of Gynecology and Gynecological Oncology, University Hospital Bonn, 14163 Bonn, Germany; Matthias.Stope@ukbonn.de; 5 UroFors Consortium (Natural Scientists in Urological Research), German Society of Urology, 14163 Berlin, Germany

**Keywords:** testosterone, biphasic effect of androgens, protein stability

## Abstract

The androgen receptor (AR) plays a central role in prostate, muscle, bone and adipose tissue. Moreover, dysregulated AR activity is a driving force in prostate cancer (PCa) initiation and progression. Consequently, antagonizing AR signalling cascades via antiandrogenic therapy is a crucial treatment option in PCa management. Besides, very high androgen levels also inhibit PCa cells’ growth, so this effect could also be applied in PCa therapy. However, on the molecular and cellular level, these mechanisms have hardly been investigated so far. Therefore, the present study describes the effects of varying androgen concentrations on the viability of PCa cells as well as localization, transactivation, and protein stability of the AR. For this purpose, cell viability was determined via WST1 assay. Alterations in AR transactivity were detected by qPCR analysis of AR target genes. A fluorescent AR fusion protein was used to analyse AR localization microscopically. Changes in AR protein expression were detected by Western blot. Our results showed that high androgen concentrations reduce the cell viability in LNCaP and C4-2 cell lines. In addition, androgens have been reported to increase AR transactivity, AR localization, and AR protein expression levels. However, high androgen levels did not reduce these parameters. Furthermore, this study revealed an androgen-induced increase in AR protein synthesis. In conclusion, inhibitory effects on cell viability by high androgen levels are due to AR downstream signalling or non-genomic AR activity. Moreover, hormonal activation of the AR leads to a self-induced stabilization of the receptor, resulting in increased AR activity. Therefore, in clinical use, a therapeutic reduction in androgen levels represents a clinical target and would lead to a decrease in AR activity and, thus, AR-driven PCa progression.

## 1. Introduction

The androgen receptor (AR), also known as NR3C4, is coded by the *AR* gene on the X chromosome (Xq11-12) [1]. The protein belongs to the nuclear receptor superfamily and is activated by binding androgenic hormones, including testosterone and dihydrotestosterone (DHT) [2,3]. The pivotal role of the AR includes differentiation of the prostatic luminal epithelial cells and regulation of gene expression which is necessary for prostate function, growth, and survival [4]. Besides, the AR maintains muscle, bone, and adipose tissue [5]. The AR protein structure is similar to other nuclear receptors [6,7,8]. It consists of eight exons sequentially coding the N-terminal transactivation domain, the DNA-binding domain, the hinge region, and the ligand-binding domain [6,7,8]. The inactive AR is associated with heat-shock proteins (e.g., HSP40, HSP70, and HSP90) in the cytoplasm [9,10]. Once androgens are taken up into the cell, they are converted to the active form DHT by 5α-reductase. DHT-mediated activation of the AR causes a conformational change where the AR dimerizes, which triggers AR nuclear translocation, followed by binding to androgen response elements (ARE) of the DNA. After binding to the ARE, coregulators are recruited by the AR in a ligand-dependent manner to either enhance (coactivator, e.g., CBP) or repress (corepressor, e.g., NCoR) the AR target gene transcription [11,12]. Coregulators mediate the AR’s ability to transactivate the target gene, such as the prostate-specific antigen (PSA, also known as Kallikrein 3) or the transmembrane serine protease (TMPRSS2) through chromatin remodelling and basal transcriptional machinery recruitment [11,12,13]. Genes regulated by the AR modify protein folding, trafficking and secretion, cell cycle, metabolism, biosynthetic pathways, and regulation of several transcription factors [13].

The AR is involved in various disorders, including androgen insensitivity syndrome, spinal bulbar muscular atrophy, hypogonadism, benign prostatic hyperplasia, and prostate cancer (PCa) [10,14,15,16,17,18].

According to the “Cancer Today” project, PCa has the second-highest incidence of all cancers in men, with 1,414,259 new diagnosed cases worldwide and 375,304 PCa-related deaths yearly [19]. As proliferation and progression of PCa are highly dependent on androgens, androgen deprivation therapy (ADT) and treatment with antiandrogens are the gold standard for locally advanced or metastatic PCa [20]. Since 2006, it is also discussed that, next to the inhibition of the AR by ADT and antiandrogens, treatment with high testosterone levels also inhibits prostate cancer proliferation and may provide a new therapeutic option [21].

This study aimed to investigate changes in the AR signalling after treatment with low and high androgen concentrations to identify possible concentration-dependent differences. To this end, the two AR-positive cell lines (LNCaP and C4-2) and one AR negative cell line (PC3) have been treated with different concentrations of the synthetic androgen R1881 (Table 1). Following the R1881 treatment, the influence on cell viability, AR transactivation, AR localisation, and AR stability has been investigated.

## 2. Materials and Methods

### 2.1. Cell Culture

The human PCa cell lines LNCaP and PC3 were obtained from the American Type Culture Collection (ATCC). Prof. Thalmann (University of Berne, Berne, Switzerland) kindly provided the C4-2 cell line [23]. LNCaP and its subcell line C4-2 represent a castration sensitive (LNCaP) and castration-resistant (C4-2) phenotype. LNCaP, C4-2, and PC3 were cultured as described in Erb et al., 2020 [26]. The main characteristics of the used cell lines are listed in Table 1. Mycoplasma testing was performed using the Mycoalert Detection Assay (Lonza, Basel, Switzerland). STR profiling was used to verify cell line authentication. For the experiments, steroid hormone and growth factors withdrawal were achieved by a medium change to 5% dextran-coated charcoal treated FBS (FBSdcc; Thermo Fischer Scientific, Waltham, MA, USA). Androgen treatment was performed using the synthetic androgen R1881 (Sigma-Aldrich, St. Louis, MO, USA, R0908-10MG, Lot No: 085M4610V) dissolved in DMSO (20 µM stock solution), and aliquots were stored at −80 °C.

### 2.2. WST-1 Cell Viability Assay

Cell viability was analysed using the Cell Proliferation Reagent WST-1 (Roche, Mannheim, Germany). Cells (10,000 cells in 100 μL) were seeded into 96-well culture plates. After 24 h, the medium was subsequently changed to a 50 µL medium containing 5% FCSdcc (Thermo Fisher Scientific, Waltham, MA, USA) for 24 h. After another 24 h, cells were treated with different concentrations of R1881 (0.01–10 nM). After 72 h incubation, 10 µL WST-1 solution was added for an additional 2 h. Absorbance was measured at 450 nm (Reference 620 nm) using Mithras LB 940 (Berthold Technologies, Bad Wildbad, Germany). After subtracting background absorbance, results were displayed as x-fold of untreated cells.

### 2.3. Total RNA Isolation, cDNA Synthesis, and Quantitative Real-Time PCR (qPCR)

Total RNA was isolated using the DIRECT-ZOL RNA MINIPREP (Zymo Research, Freiburg im Breisgau, Germany) according to the manufacturer’s instructions. Superscript II RNase H Reverse Transcriptase kit was used for cDNA synthesis with 500 ng total RNA (Thermo Fisher Scientific). qPCR was performed with GoTaq Probe qPCR Master Mix (Promega, Mannheim, Germany) and appropriate primers for 45 cycles on a LightCycler 480 instrument (Roche, Mannheim, Germany). The geometric mean of HPRT1 and TBP was used for normalisation. The The LightCycler^®^480 Software, Version 1.5 (Roche, Mannheim, Germany) was used to determine crossing point (Cp) values. Delta (Δ)Cp = Cp_GOI_ − Cp_Housekeeper_ values were calculated and expressed as relative mRNA expression (2^−ΔCp^). The following primer assays have been used (all Thermo Fisher Scientific, Waltham, MA, USA): *AR* (Hs00171172_m1), *PSA/KLK3* (Hs02576345_m1), *TMPRSS2* (Hs01122322_m1), *PROSTEIN/SLC45A3* (Hs01026319_g1), HPRT1 (Hs02800695_m1), and TBP (Hs00427620_m1).

### 2.4. Western Blot Analysis

Cells (400,000 cells per well) were seeded into 6-well culture plates for Western blot analysis. After 24 h, the medium was changed to a medium containing 5% FCSdcc (Thermo Fisher Scientific, Waltham, MA, USA). After another 24 h, cells were treated with different concentrations of R1881 (0.01–10 nM) for 72 h. Subsequently, cells were harvested, and protein concentration determination was performed as previously described [26]. NuPAGE^TM^ 4–12% Bis-Tris protein gels separated 20 µg protein lysate. As protein standard, 5 µL Spectra Multicolour Broad Range (Thermo Fisher Scientific, Waltham, MA, USA) protein standard mixed with 1 µL MagicMark^TM^ XP Western Protein Standard (Thermo Fisher Scientific, Waltham, MA, USA) were used. Proteins were transferred to a nitrocellulose membrane using the iBlot Dry Blotting System (all Thermo Fischer Scientific, Waltham, MA, USA). WesternBright Sirius HRP substrate (Advansta, San Jose, CA, USA) was used to detect signals and digitalised using a Microchemi chemiluminescence system (DNR Bio-Imaging Systems, Ha-Satat, Israel). The antibodies used are listed in Table 2. Experiments were analysed with the Image-Studio Lite 5.2 software (LI-COR, Lincoln, NE, USA).

### 2.5. Immunofluorescence

Cells (50.000 per chamber) were seeded into 8 well chamber slides and incubated for 24 h. After 24 h, the medium was changed to FCSdcc for another 24 h. For localisation studies, cells were subsequently treated with different concentrations of R1881 (0.01–10 nM) for 2 h. First, the cells were washed with PBS for staining and fixed with 4% paraformaldehyde for 10 min, followed by three times 10 min washing with PBS. Then, to permeabilise the cells, 300 µL PBS + 1% bovine serum albumin (BSA) + 0.2% Triton X100 was pipetted in each chamber for 5 min at 4 °C. The permeabilisation step was followed by a 30 min blocking step with PBS + 1% BSA. For antibody incubation, cells were incubated for 24 h with primary antibodies at 4 °C (Androgen Receptor (D6F11) XP Rabbit mAb, Cell Signaling Technology, Danvers, MA, USA, Dilution: 1:500). After 3 × 10 min washing with TBS-T, cells were incubated with the fluorescence-labelled secondary antibodies goat anti-rabbit 488 (Invitrogen, Dilution: 1:1000) and the DNA staining dye Hoechst 33342 (Abcam, Dilution: 1:1000) for 1 h. After 10 min washing with TBS-T, followed by 10 min washing with TBS, and 10 min washing with deionised H_2_O, chamber slides were finally mounted with Vectashield Hard Set mounting medium containing DAPI (Vector Laboratories, Burlingame, CA, USA). The cells were visualised using a Compact Fluorescence Microscope BZ-X800E (Keyence, Osaka, Japan). CellProfiler 4.2.1 software has been used to calculate the nuclear/cytoplasmic intensity ratio and to measure total AR intensity [27].

### 2.6. eosFP-AR Transfection

pAR-t1EosFP, a pCDNA3 expression vector coding for the AR fusion protein AREos, was provided by Dr. Marcus Cronauer (Institute of Pathology, Bonn, Germany) and Dr. F. Oswald (Klinik für Innere Medizin III, Ulm, Germany) [28,29]. The plasmid is coding a green fluorescent AR fusion protein controlled by a CMV promotor [28,29]. The EosFP is a photoactivatable green (516 nm) fluorescent protein able to switch to red fluorescence upon UV irradiation (~390 nM) [30]. For transfection experiments, 2 μg/mL plasmid in six-well plates with the ViaFect™ Transfection Reagent (Promega, Mannheim, Germany) were used for 24 h according to the manufacturer’s instructions. Following the transfection, the cells were starved for 24 h using 5% FCSdcc. Subsequently, the cells were treated with different concentrations R1881. The cells were visualised using IncuCyte^®^ S3 Live-Cell Analysis (Essen BioScience, Newark, UK).

### 2.7. Statistical Analysis

Prism 9.3 (GraphPad Software. GraphPad Software, San Diego, MA, USA) was used for statistical analysis. Differences between treatment groups were analysed using Student’s *t*-test or ordinary one-way ANOVA. Data are presented as mean ± s.e.m. or mean ± s.d. to estimate the various means in multiple repeated experiments [31]. *p*-values of ≤0.05 were considered statistically significant. All differences highlighted by asterisks were statistically significant (* *p* ≤ 0.05; ** *p* ≤ 0.01; *** *p* ≤ 0.001).

## 3. Results

### 3.1. High R1881 Concentrations Inhibit the Cell Viability in LNCaP Cells

**Figure 1:**
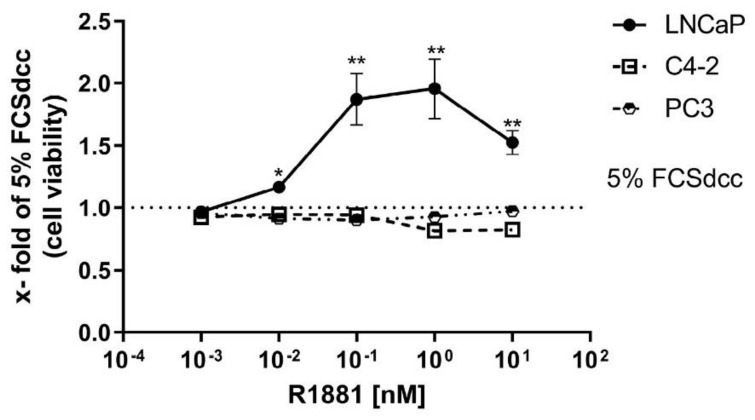
High R1881 concentrations inhibit the cell viability in LNCaP cells: Relative change of cell viability after 72 h of untreated and R1881 (0.01, 0.1, 1, 10 nM) treated LNCaP, C4-2, and PC3 cells. LNCaP cells show a concentration-dependent increase of cell viability, peaking between 10^−1^ (0.1) and 10^0^ (1) nM after R1881 treatment. Conversely, 10 nM R1881 can inhibit the cell viability increasing effects in LNCaP cells. At 1 and 10 nM, C4-2 cells show a decrease to ~80% of the untreated condition. Cell viability does not change in PC3 cells after R1881 treatment. Values are expressed as x-fold of untreated cells and are displayed as mean ± s.e.m. (*n* = 3). All differences highlighted by asterisks were statistically significant (* *p* ≤ 0.05, ** *p* ≤ 0.01).

To assess the influence of different concentrations of different levels of R1881 (0, 0.001, 0.01, 0.1, 1, 10 nM) on prostate cancer cell lines, the WST-1 cell viability assay was performed on LNCaP, C4-2, and PC3 cells after R1881 treatment (Figure 1). Increasing concentrations of R1881 caused a concentration-dependent increase in cell viability, with the highest cell viability found between 0.1 (10^−1^) to 1 (10^0^) nM. Compared to the cell viability at 1 nM, cell viability was reduced at a concentration of 10 nM R1881 in LNCaP cells (Figure 1). In contrast, C4-2 cells showed no change in cell viability up to 1 nM. At 1 and 10 nM, C4-2 cells showed a decrease down to ~80% of the untreated condition. PC3 cells did not show any change in cell viability after R1881 treatment.

### 3.2. High R1881 Concentrations Do Not Inhibit AR-Mediated Gene Transcription

**Figure 2:**
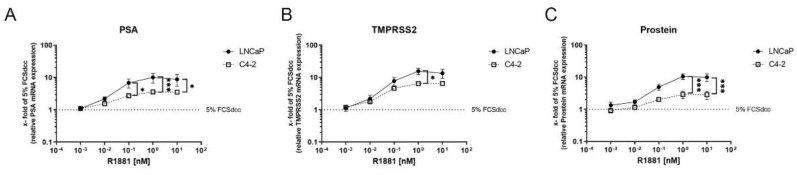
High R1881 concentrations do not inhibit AR-mediated gene transcription: To determine AR-mediated gene transactivation, modulation of mRNA levels of three AR target genes (PSA, TMPRSS2, PROSTEIN) were detected by qPCR and displayed as a mean change of untreated condition (5% FCSdcc, dotted line). (**A**–**C**) Relative change of the AR target genes PSA (**A**), TMPRSS2 (**B**), and PROSTEIN (**C**) after 16 h of different concentrations of R1881 in LNCaP and C4-2 cells. Values are expressed as x-fold of untreated cells (5% FCSdcc, dotted line) and are displayed as mean ± s.e.m. (*n* = 3). All differences highlighted by asterisks were statistically significant (* *p* ≤ 0.05, *** *p* ≤ 0.001).

Change in gene transcription of AR target genes can be used as surrogates for AR-mediated transactivation. Therefore, changes in the AR target genes PSA, TMPRSS, and Prostein have been measured after treatment with different R1881 concentrations (0.01, 0.1, 1, 10 nM). All tested AR targets showed a concentration-dependent increase after R1881 treatment in both cell lines (Figure 2A–C). The increase reached a plateau in all cell lines at 1 nM and showed no further change at 10 nM. Comparison of the cell lines revealed a significantly stronger increase in the tested AR targets in LNCaP cells.

### 3.3. R1881 Activates Nuclear AR Translocation in a Time and Concentration-Dependent Manner

**Figure 3:**
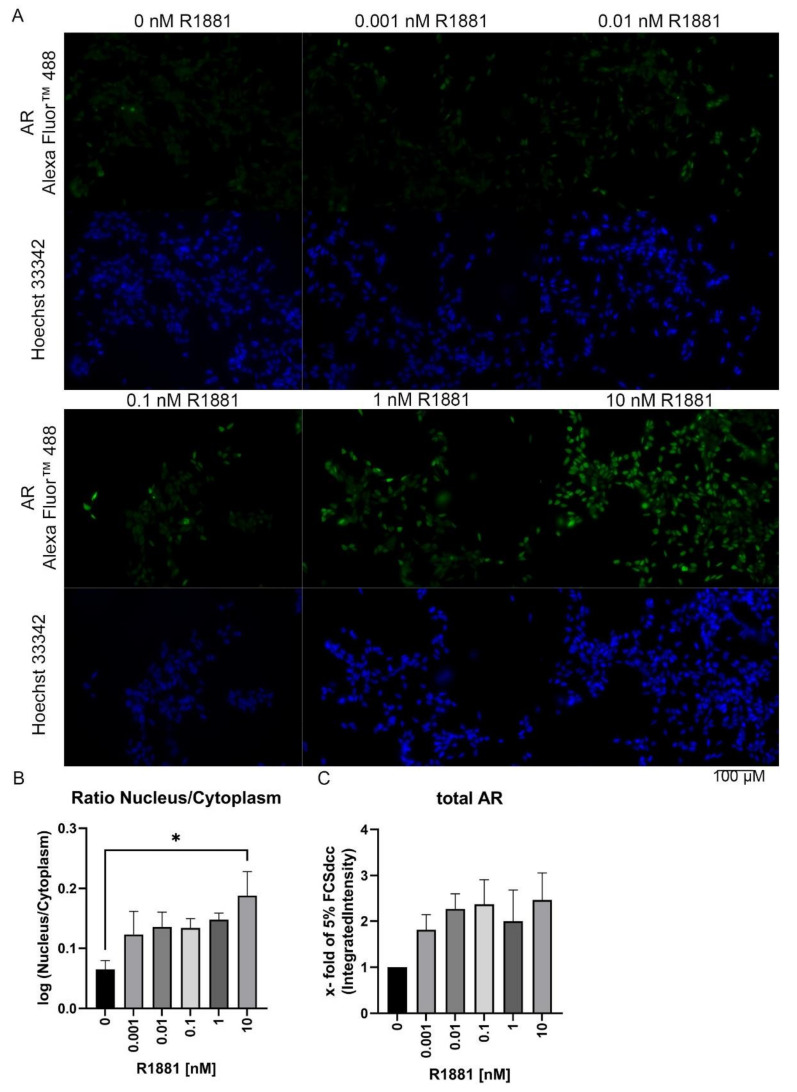
R1881 increases nuclear localisation in a concentration-dependent manner: (**A**) Representative Immunofluorescence staining of R1881 (0–10 nM) treated LNCaP cells. Scale bar represents 100 µM. (**B**) Nuclear to cytoplasmic ratio after 2 h of R1881 treatment in LNCaP cells. (**C**) Relative change of total AR after 2 h of R1881 treatment. Values are expressed as x-fold of untreated cells and are displayed as mean ± s.d. (*n* = 3). All differences highlighted by asterisks were statistically significant (* *p* ≤ 0.05).

To assess the influence of different concentrations of R1881 on AR localisation, PC3 cells were transfected with the fusion protein eosFP-AR (Figure S1, Figure S2 and Figure S3A,B). Subsequently, the AR fusion protein was tracked after R1881 treatment using the IncuCyte^®^ S3 Live-Cell Analysis live-cell imager. Image analysis revealed a concentration and time-dependent increase in nuclear AR protein (Figure S3B). At 10 nM R1881, all cells have a nuclear AR localisation after 15 min. However, at 0.001 nM, only 15% of the cells showed a nuclear AR localisation after 24 h (1440 min). To validate this finding, LNCaP cells were treated with different R1881 concentrations for 2 h and AR localisation was assessed using immunofluorescence (Figure 3A). In line with the results obtained from the fusion protein experiments, the R1881 treatment causes an increase in nuclear AR protein (Figure 3B), peaking at 10 nM. Moreover, the R1881 treatment led to a rise in total AR levels (Figure 3C).

### 3.4. AR Is Stabilised Transcriptionally Independent by R1881

**Figure 4:**
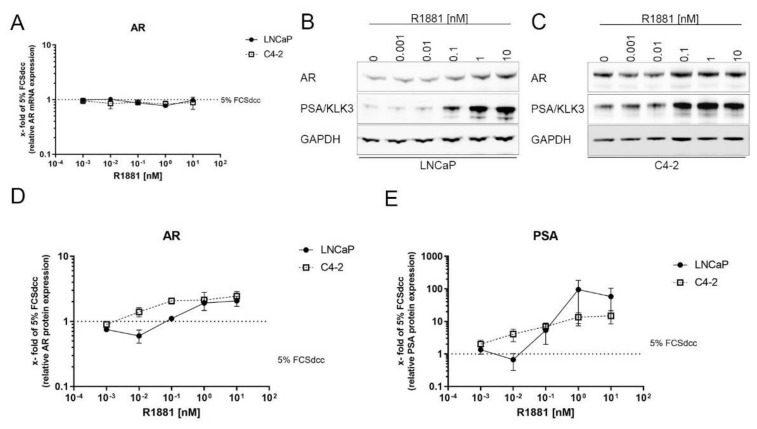
R1881 changes AR protein in a transcription-independent way: (**A**) Relative change of AR mRNA levels after 16 h treatment with increasing concentrations of R1881 in LNCaP and C4-2 cells. Values are expressed as x-fold of untreated cells and are displayed as mean ± s.e.m. (*n* = 3). (**B**,**C**) Representative Western Blot of 72 h R1881 treated LNCaP (**B**) and C4-2 (**C**) cells. (**D**) Relative change of AR protein in LNCaP and C4-2 cells after 72 h treatment with increasing concentrations of R1881. Values are expressed as x-fold of untreated cells and are displayed as mean ± s.d. (*n* = 3). (**E**) Relative change of the treatment response PSA in LNCaP and C4-2 cells after 72 h treatment with increasing concentrations of R1881. Values are expressed as x-fold of untreated cells and are displayed as mean ± s.d. (*n* = 3). Uncropped Western blots are displayed in Figure S4.

The immunofluorescence experiments indicate an increase in total AR protein after R1881 treatment in LNCaP cells (Figure 3C). To investigate if R881 induced a change in *AR* mRNA, LNCaP and C4-2 cells were treated for 16 h with increasing concentrations of R1881 and changes in *AR* mRNA were assessed using qPCR. Treatment with R1881 did not cause any change in *AR* mRNA levels after 16 h (Figure 4A). However, treatment with R1881 led to increased concentration-dependent the AR protein levels (Figure 4B–D). In LNCaP, the increase of AR protein reached a plateau at 1 nM R1881. In C4-2 cells, the AR protein increase reached a plateau at 0.1 nM R1881. In addition, the treatment control PSA increased concentration-dependent peaking at 1 nM for LNCaP and showed the highest expression at 10 nM in C4-2 (Figure 4E).

### 3.5. Inhibition of the Protein Synthesis by Cycloheximide Prevents R1881 Induced AR Protein Increase

**Figure 5:**
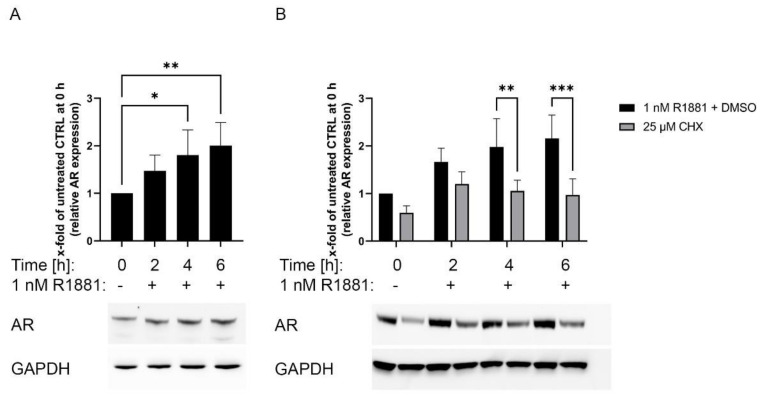
Cycloheximide inhibits R1881 induced AR protein increase: (**A**) Relative change and representative Western blot of 1 nM R1881 treated LNCaP cells after 2,4, and 6 h. (**B**) Relative change and representative Western blot of 1 nM R1881 treated LNCaP cells combined with DMSO (vehicle control) or 25 µM Cycloheximide (CHX) in LNCaP cells after 2,4, and 6 h. Values are expressed as x-fold of untreated cells and are displayed as mean ± s.d. (*n* = 3). All differences highlighted by asterisks were statistically significant (* *p* ≤ 0.05, ** *p* ≤ 0.01, *** *p* ≤ 0.001). Uncropped Western blots are displayed in Figure S5.

This study’s fluorescence and Western blot results indicated that R1881 increased AR protein in a transcriptional independent manner (Figure 3C and Figure 4). Therefore, the influence of R1881 on AR mRNA and protein levels was assessed after 2, 4, and 6 h of R1881 treatment. On mRNA level, AR expression was not changed after 1 nM R1881 in the first 6 h after treatment as revealed by the public available dataset of Massie et al. (Figure S5A) [32]. However, Western blot analysis revealed that R1881 significantly increased AR protein after 4 h (Figure 5A). The R1881 induced increase in AR protein (Figure 5B) can be significantly inhibited by the simultaneous treatment with 25 nM of the protein synthesis inhibitor cycloheximide (CHX).

## 4. Discussion

The AR signalling has been reported to play an essential role in several diseases, including PCa [10,14,15,16,17,18]. Furthermore, treatment with high levels of androgens has been discussed as a possible therapeutic option in PCa [21]. Therefore, the influence of low and high levels of androgens on AR signalling have been investigated in this study. Multiple investigations have reported decreased cell proliferation of the hormone-sensitive LNCaP cell line when treated with concentrations above 1 nM R1881 [21,33]. In detail, optimal growth media supplemented with 10 nM R1881 induced cell growth inhibition, cell cycle arrest and apoptosis in LNCaP cells [21,33].

Additionally, here, the reported biphasic effect of R1881 could be confirmed in LNCaP cells, but to a smaller extent as reported before [33,34,35]. Additionally, the CRPC cell line model C4-2 reduced cell viability at high concentrations. However, in contrast to the castration-sensitive LNCaP cells, the androgen deprivation and R1881 treatment up to 1 nM did not affect cell viability in C4-2 cells. This difference between the cell lines is based on the castration resistance of the C4-2 cell line [23]. Literature has reported that the AR splice variant V7 as expressed in C4-2 cells is responsible for a constitutive AR activation and androgen-independent cell growth [36,37,38]. However, adaptions to high levels of R1881 have also been reported previously and explained by extensively passaging [21]. Several mechanisms have been introduced to be responsible for the biphasic effects of AR, including transcription of cell cycle-inhibitory genes like *p16* and *p21*, downregulation of MYC, *CDK1*, and *BCL2*, and induction of oxidative stress [39,40,41,42,43]. Moreover, it is suggested that the biphasic effect is partially explained by the influence of R1881 concentrations on coactivators influencing the transcriptional activity of nuclear receptors [33,44]. Therefore, the influence of low and high R1881 concentrations on the AR target genes *PSA*, *TMPRSS2*, and *PROSTEIN* was assessed. Both tested cell lines showed a concentration-dependent increase of the three AR target genes *PSA*, *TMPRSS2*, and *PROSTEIN*, reaching a plateau at 1 nM R1881. In line with previous observations, this result shows no change in AR-mediated gene transcription above 1 nM R1881 [43]. This observation indicates that supraphysiological androgens do not suppress androgen receptor-mediated gene transcription.

In addition, a high concentration of R1881 had no inhibitory effects on AR localisation. However, it could be shown that high R1881 levels accelerate AR translocation to the nucleus. Moreover, the localisation study indicates an increase in AR protein levels after R1881 treatment.

Increased AR protein after androgen treatment has previously been described in several studies [45,46,47,48]. This increase of AR protein after R1881 treatment is concentration-dependent, reaching a plateau at 1 nM and showing no further change at higher concentrations. In contrast, mRNA expression analysis of the Massie et al. dataset revealed no increase in AR mRNA levels after treatment with R1881 in the first 6 h after treatment. As this regulation is independent of changes in AR transcription and starts after 2 h, there is strong evidence that androgens influence AR stability or AR protein turnover. Changes in AR stability after inhibition of the AR signal pathway have been reported previously in an independent proteasome mechanism [47,49,50]. In addition, high levels of PIAS1 and STAT5 enhance AR protein stability after androgen deprivation or antiandrogen treatment and therefore influence AR transactivity [26,50]. Moreover, the AR protein is stabilised by HSPs, thus affecting the antiandrogen efficiency [51]. Glycogen-Synthase Kinase-3 has also been reported to influence AR protein stability in LNCaP and 22Rv1 cells [28]. Mora and colleagues reported protein synthesis-dependent regulation of AR protein after androgen treatment [46].

Additionally, here, a protein synthesis-dependent regulation of AR protein after R1881 treatment could be shown. As this increase in AR protein can already be seen after 2 h and is reversed by the protein synthesis-inhibitor CHX, there is strong evidence that non-genomic AR activity is responsible for AR stabilisation. The influence of AR on protein synthesis has already been described before [52,53,54]. It has been reported that castrated *PTEN ^L/L^* mice exhibit a 30% increase in de novo protein synthesis per cell compared to non-castrated mice [52]. It is suggested that the AR interacts and influences the eIF4F complex, a critical driver of protein synthesis. Another study has shown that eIF2α has been phosphorylated by activated AR and subsequently promotes endogenous *TMEFF2* translation [54]. Together with the observations of this study, these reports suggest that increased AR protein levels are due to an increase in AR protein syntheses, possibly triggered by a non-genomic AR effect.

## 5. Conclusions

This study investigated the influence of low and high concentrations of androgens on parts of the AR signalling cascade. As already reported in the literature, it could be shown that high concentrations lead to reduced cell viability of LNCaP. However, high androgen concentrations do not negatively affect AR-mediated gene expression, nuclear localisation, and AR protein stability. Therefore, the reported negative effects may be due to AR downstream targets and pathways. In addition to these results, this study demonstrated that androgen-dependent activation of the AR increases AR protein synthesis. Therefore, it can be assumed that the AR stabilises itself after activation, thus positively influencing its activity, representing a possible therapeutic target for PCa.

## Figures and Tables

**Table 1:** Characteristics of the used cell lines.

Name	Characteristics	AR Status	Origin	References
LNCaP	Androgen dependent	AR T877A	Lymph node	[22]
C4-2	Androgen independent	AR T877A	Lymph node	[23]
PC3	small cell neuroendocrine carcinoma	AR negativ	Bone metastasis	[24,25]

**Table 2:** Antibodies used in the study.

Name	Company	Lot	Dilution
Androgen Receptor (D6F11) XP Rabbit mAb	Cell Signaling Technology	9	1:5000
Mouse Monoclonal anti-GAPDH (6C5)	Novus Biologicals	19/05-G4cc-C5cc	1:10,000
Polyclonal Rabbit Anti-Mouse Immunoglobulins/HRP	Agilent Technologies	20066043	1:10,000
Polyclonal Swine Anti-Rabbit Immunoglobulins/HRP	Agilent Technologies	41289300	1:10,000

## Data Availability

The data presented in this study are available in this article and Supplementary Materials.

## Supplementary Materials

The following are available online at https://www.mdpi.com/article/10.3390/cimb44020041/s1, Figure S1: Representative Picture of eosAR experiments Part 1. The figures show representative pictures of eosAR transfected PC3 cells treated with 0 and 0.01 nM R1881 at different timepoints; Figure S2: Representative Picture of eosAR experiments Part 2. The figures show representative pictures of eosAR transfected PC3 cells treated with 0.1 and 1 nM R1881 at different timepoints; Figure S3: (**A**) Representative Picture of eosAR experiments Part 3. The figures show representative pictures of eosAR transfected PC3 cells treated with 10 nM R1881 at different timepoints. (**B**) Evaluation of the eosAR experiments (**C**) Specify controls of Immunofluorescence; Figure S4: Uncropped Western blots of Figure 4; Figure S5: Uncropped Western blots of Figure 5.
