# Supplementary material for: The Androgen Hormone-Induced Increase in Androgen Receptor Protein Expression Is Caused by the Autoinduction of the Androgen Receptor Translational Activity
Source: Curr Issues Mol Biol. 2022 Jan 25;44(2):597–608. doi: 10.3390/cimb44020041 (PMC8928990; doi:10.3390/cimb44020041)

Figure S1: Representative Picture of eosAR experiments Part 1

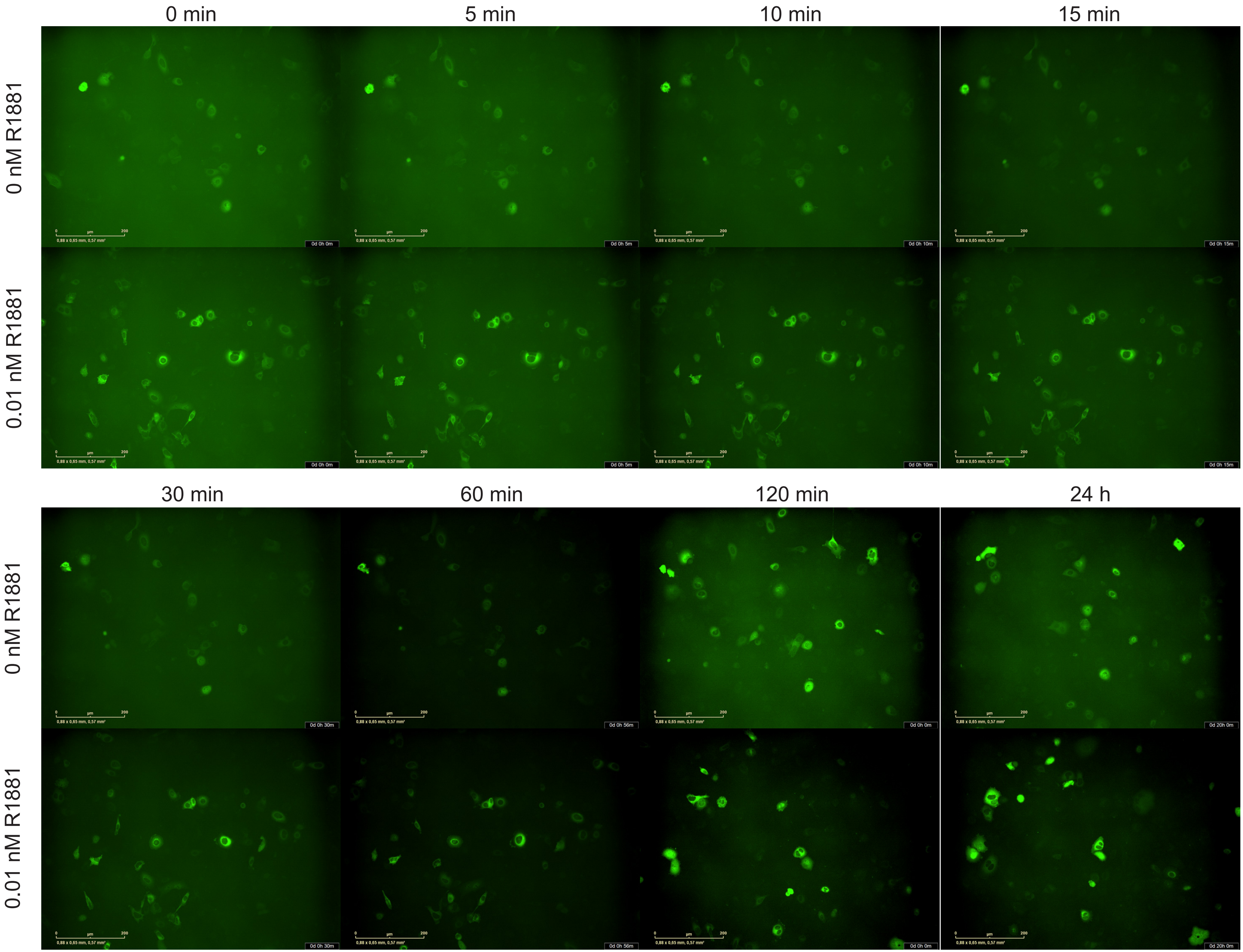

Figure S2: Representative Picture of eosAR experiments Part 2

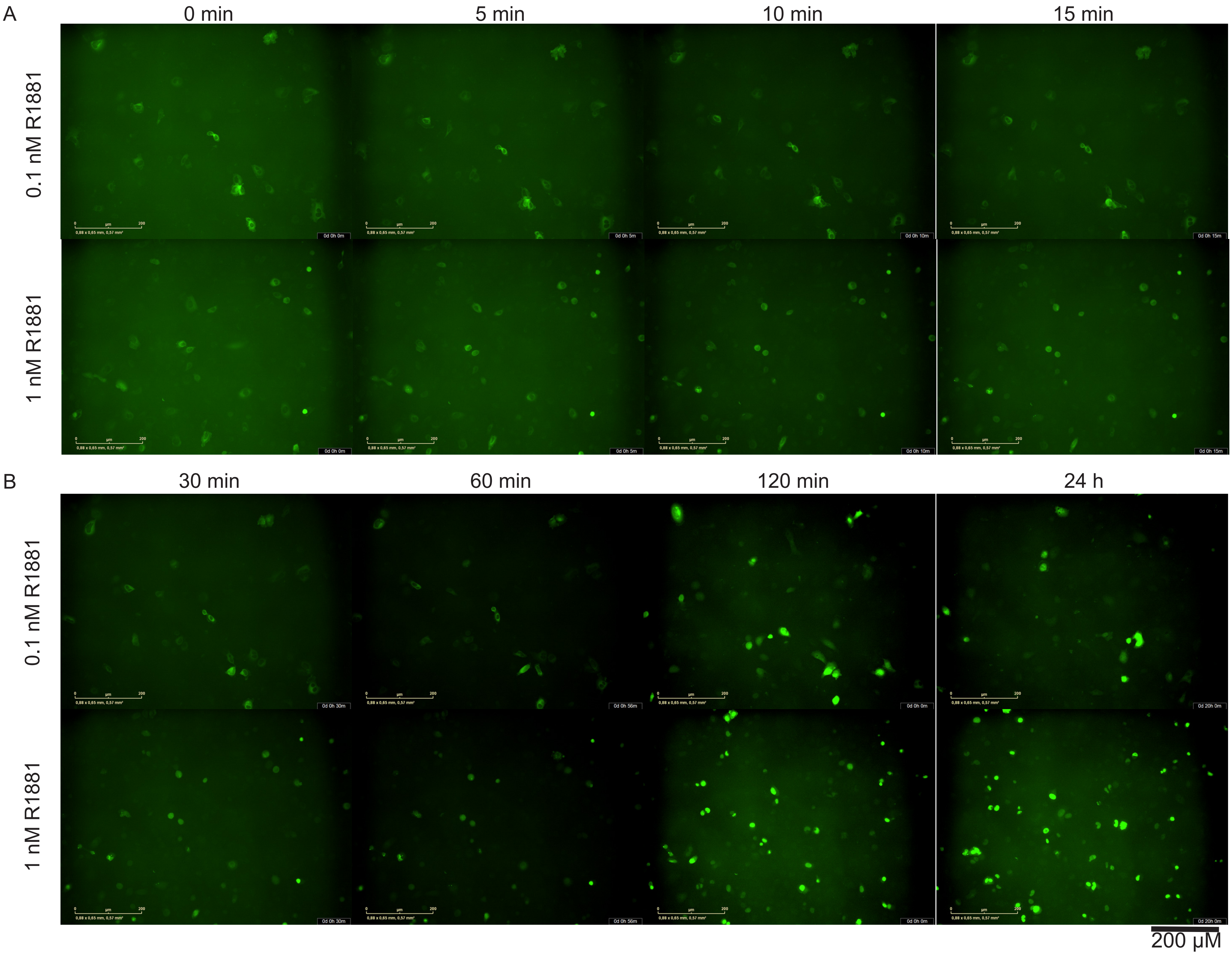

Figure S3: : (A) Representative Picture of eosAR experiments Part 3 (B) Evaluation of the eosAR experiments (C) Specify controls of Immunofluorescence

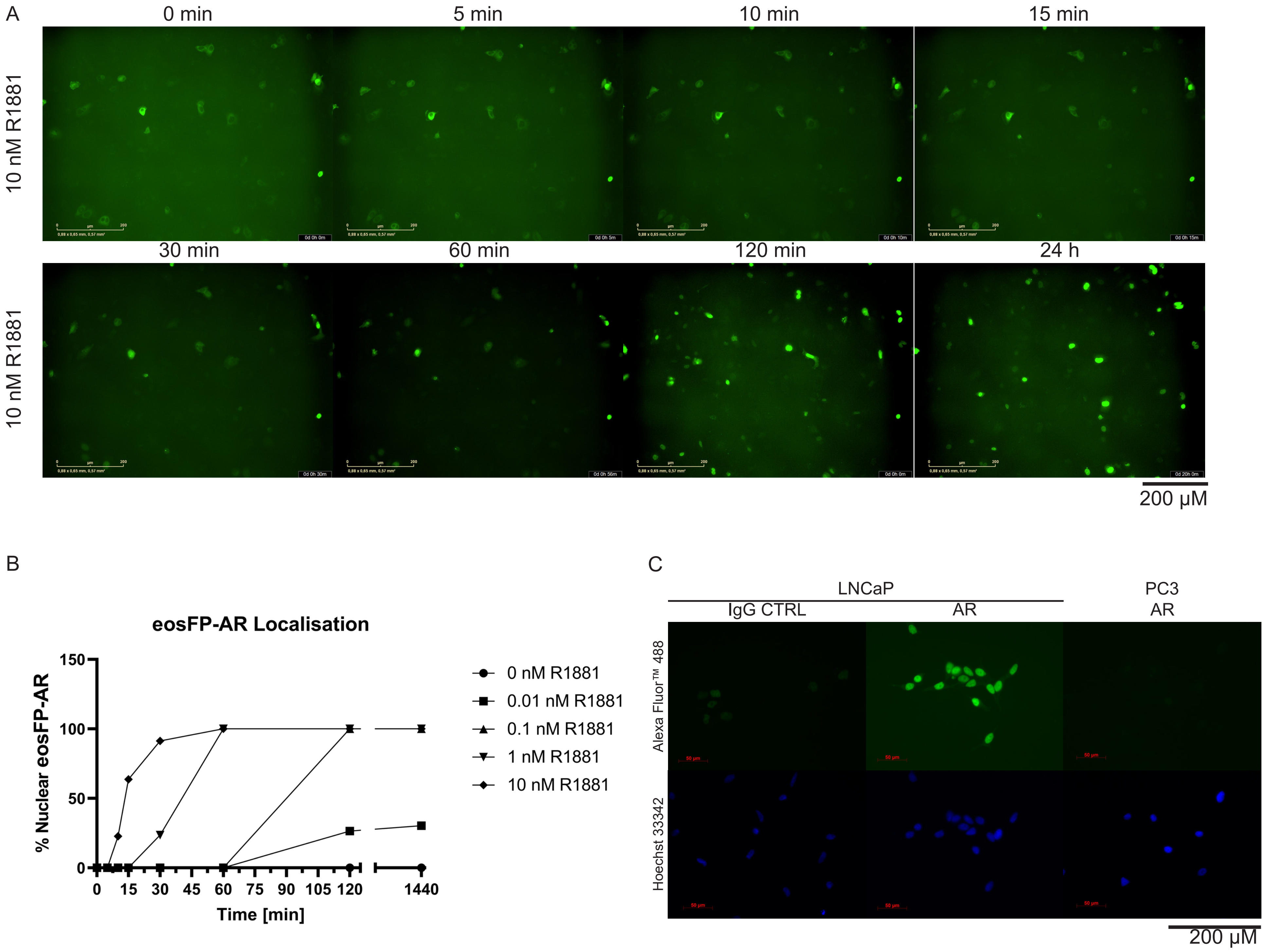

Figure S4: Uncropped Western blots of Figure 4

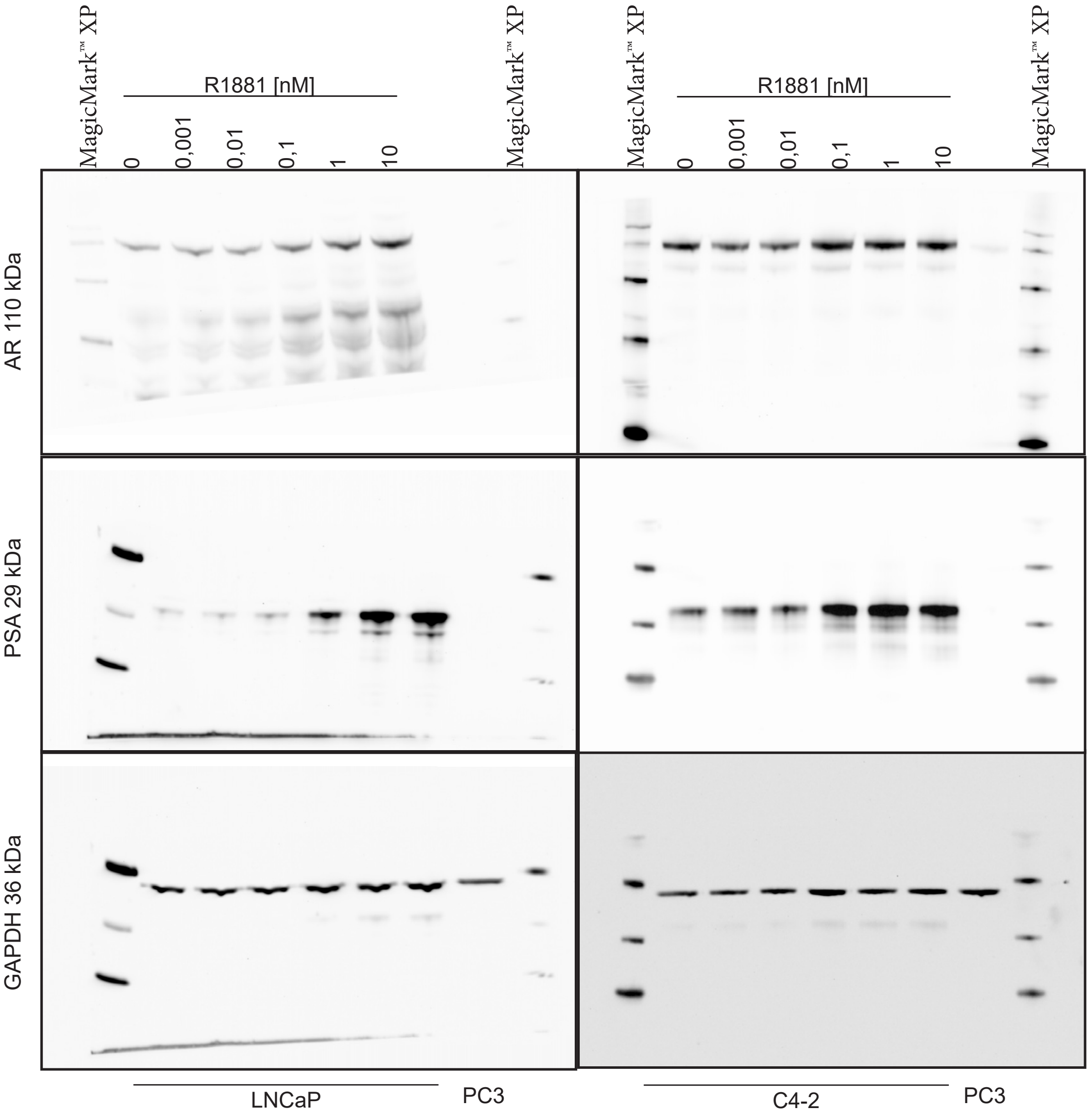

Figure S5: Uncropped Western blots of Figure 5

A

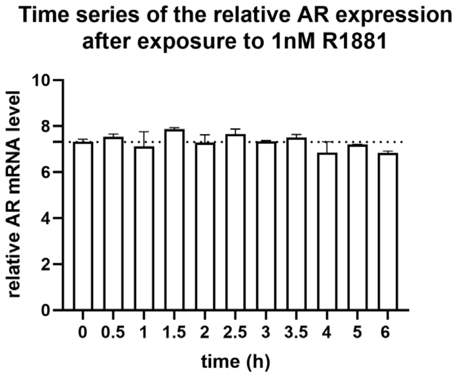

B

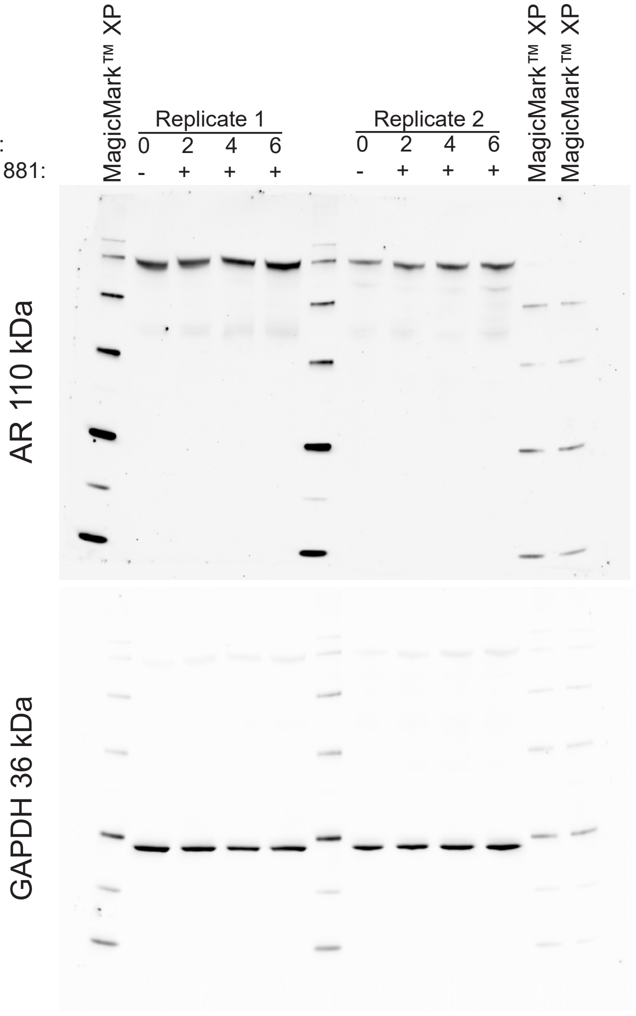

C

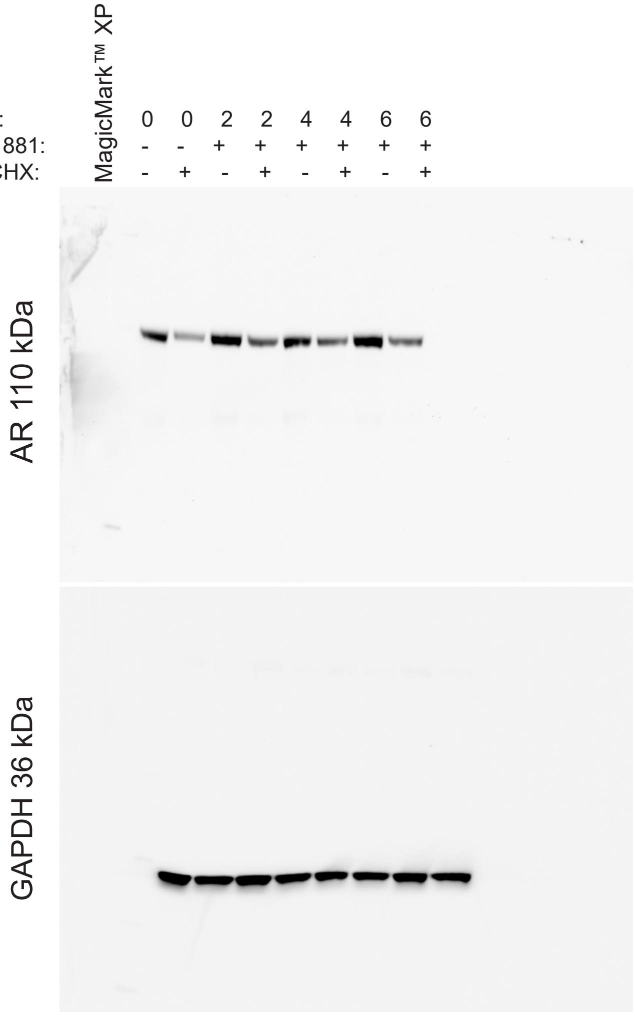

Supplement: Supplementary file 1 [file cimb-44-00041-s001.zip › cimb-1545222-supplementary.pdf]
